# Distribution Characteristics of Indoor PM_2.5_ Concentration Based on the Water Type and Humidification Method

**DOI:** 10.3390/ijerph17228638

**Published:** 2020-11-20

**Authors:** Seonghyun Park, Janghoo Seo, Sunwoo Lee

**Affiliations:** 1 Department of Industry-Academic Cooperation Foundation, Kookmin University, 77, Jeongneung-ro, Seongbuk-gu, Seoul 02707, Korea; marine86@kookmin.ac.kr; 2 School of Architecture, Kookmin University, 77, Jeongneung-ro, Seongbuk-gu, Seoul 02707, Korea; 3 Department of Construction Science, College of Architecture, Texas A&M University, TX 77843, USA; envswlee@gmail.com

**Keywords:** humidifier, particulate matter, white dust, indoor air quality, calibration

## Abstract

With the industrialization and rapid development of technology that can measure the concentration of pollutants, studies on indoor atmosphere assessment focusing on occupants have been recently conducted. Pollutants that worsen indoor atmosphere include gaseous and particulate matter (PM), and the effects and diffusion characteristics that influence indoor atmosphere vary depending on the indoor and outdoor concentration. White dust is a PM generated from minerals in water used for humidifiers during winter. Therefore, studies on the impact of white dust on human health and its size distribution are being actively conducted. However, since the indoor PM concentration varies depending on the humidification method and water type used, relevant studies are needed. Accordingly, this study examined the change in the PM_2.5_ concentration and relative humidity on the basis of water types and humidification method. It was found that the indoor PM_2.5_ concentration varied from 16 to 350 ug/m^3^, depending on the water types used for an ultrasonic humidifier. Conversely, when using a natural evaporative humidifier, white dust did not increase the indoor PM_2.5_ concentration, regardless of the mineral content of the water used. Considering both humidification ability and continuous humidifier use indoors, water purifier with nano-trap filters must be utilized for ultrasonic humidifiers.

## 1. Introduction

Recently, indoor air quality (IAQ) has been deteriorating due to the high airtightness of buildings and the use of building materials composed of complex chemicals. As a result, various studies for the health of residents have been conducted [1,2,3]. The inflow paths of particulate matter (PM) could significantly impact indoor PM concentration, including openings for ventilation and windows that can be recognized by occupants, as well as doors and chinks in the door that are difficult to recognize [4]. Particulate pollutants consist of filterable particulate matter (FPM) and condensable particulate matter (CPM) based on emission characteristics [5]. FPM is generally emitted in solid or liquid form and can be captured by filter [6], whereas CPM is a particulate matter (PM) with a diameter generally less than 2.5 μm and is created through the physicochemical reaction of gaseous pollutants in the air [7].

FPM and CPM mixed in the outdoor air can increase the indoor PM concentration by infiltration and natural ventilation [8]. Likewise, the indoor FPM and CPM formed by various factors contribute to the increase in the indoor PM concentration. All behaviors causing the air current movement, including smoking, cooking, use of humidifiers, cleaning, and resuspension, correspond to these various factors [9,10,11].

Among these, the use of humidifiers to prevent respiratory diseases and improve the indoor thermal environment significantly contribute to the increase in the indoor PM concentration [12]. In the USA, approximately 17.2 million households use humidifiers [13]. Similarly, considering the climate in South Korea, wherein temperature and humidity are low during winter, it is believed that most Koreans use humidifiers [14]. Generally, the portable humidifier used at home has three types of humidification: ultrasonic, impeller, and nature evaporative [15]. The use of ultrasonic humidifiers causes two human health problems. One is hypersensitivity pneumonitis, which is caused by the bacteria originating from the water used for the humidifier that directly penetrate into the respiratory system, and the other is respiratory disease, which is caused by minerals in the water that are sprayed into the air [14,16].

Here, aerosol-type PMs emitted by minerals are called “white dust” by the Environmental Protection Agency (EPA) [17,18]. Umezawa et al. (2013) reported that, in an animal experiment, the white dust induced a cellular response, but no infection and damage occurred in the lungs of mice exposed to ultrasonic humidifiers using tap water for 7 to 14 days [19]. In this regard, several studies focusing on the harmfulness of ultrasonic humidifiers have been conducted [20,21].

The humidification principle of both impeller and nature evaporative is to evaporate water into the air. According to previous studies, since evaporative humidifiers only supply water and highly volatile compounds to the air, no PM is generated; however, no actual experiment has been conducted [15].

Moreover, the indoor PM concentration can vary depending on the water types used for the humidifier [22]. Sain et al. (2017) analyzed particles on the basis of the hardness of total dissolved solids (TDS) used for ultrasonic humidifiers. They reported that when using high TDS, the concentration inhaled by an occupant is high [15].

Energy reduction can increase the blowing load of the air purifier using a light-scattering type sensor that recognizes all the aerosol-type particles emitted from the humidifier as PM [23]. Here, instruments based on light scattering enable real-time measurement of particle concentrations at a significantly lower cost than the gravimetric and equivalent methods [24]. In addition, since the PM concentration can vary depending on the distance from the parts that emit moisture, various measuring points are required for the experiment [13]. In this case, minimizing errors in the measurement is necessary by unifying the measured values among the equipment. Thus, calibration of PM sensors using proper reference instruments and under the conditions of steady particle mass concentration should be performed.

Therefore, in this study, calibration was performed to unify the measured values of the PM_2.5_ concentration, and the validity of the small chamber system was examined after installation. Moreover, the PM_2.5_ concentration was analyzed on the basis of the water types used for ultrasonic and natural evaporative humidifiers, and operation methods for minimizing the white dust generated from humidifiers were suggested.

## 2. Experimental Methods and Materials

### 2.1. Experimental Space and Measuring Equipment

**Figure 1:**
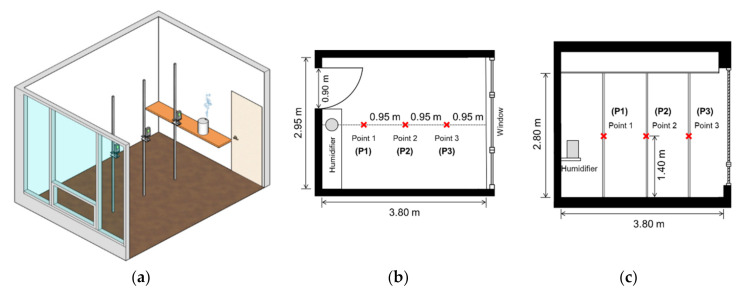
Experimental space and location of the experimental equipment: (**a**) perspective drawing of the test room; (**b**) plan of the experimental space; (**c**) section of the experimental space.

In this study, experiments were conducted to analyze the change in the indoor PM_2.5_ concentration by white dust on the basis of the water types used and humidification method. In Figure 1, the experimental space and measuring points are presented. The size of the experimental space was 3.8 × 2.95 × 2.8 m (L × W × H), 31.4 m^3^, and the humidifier was located at the center of a side wall at a height of 0.9 m from the floor. To minimize temperature deviations among the measuring points, we turned on the air conditioner located at the center of the ceiling to 24 °C in a wind-free mode for 1 h before the experiment, and the measuring equipment was initially monitored after checking whether the temperature had been properly maintained. The filter of the air conditioner was also removed before the experiment to avoid concentration reduction of the indoor PM_2.5_. In addition, when the relative humidity of the space was high before the experiment, we utilized a dehumidifier to adjust the relative humidity to less than 40%, and an air purifier was operated to keep the background PM_2.5_ concentration at 10 μg/m^3.^ or less. Blinds were used to avoid the outflow of indoor heat and inflow of insolation through the windows [13]. In this experiment, the PM_2.5_ concentration, temperature, and relative humidity were measured using the data logger during the experiment, and the measurement intervals were all set at 10 s.

For the experimental space, planned ventilation was not performed, and the space was sealed to prevent infiltration. However, the use of air conditioner to maintain a constant temperature can result in unbalanced PM_2.5_ concentrations by changing the air current distribution for each space. Therefore, the PM_2.5_ concentration was measured on the basis of various divided measuring points. As presented in Table 1, 1 AM510 unit (TSI Inc. Shoreview, MN, USA) and 3 TES-5322 units (TES Co. Taipei, Taiwan) were used to check the PM_2.5_ concentration emitted from the humidifier. Both models use the light-scattering method to measure the indoor PM concentration, but the costs differ by more than 10 times. While AM510 is already used by numerous studies owing to its verified accuracy and reliability, TES-5322 has not been frequently used despite its economic efficiency [25,26,27,28]. The measurement accuracy of TES-5322 is below ±5 μg/m^3^ when the PM_2.5_ concentration is less than 50 μg/m^3^ and below ± 10% when the PM_2.5_ concentration is greater than 50 μg/m^3^.

The precision and accuracy of all equipment must be known in order to ensure the reliability of the research results using relevant equipment or mixed models. Therefore, in this study, calibration was performed to find the reliability of the concentration measurements for each PM measuring equipment.

Table 2 presents the information of the humidifiers used in this study. Among the portable humidifiers, ultrasonic and evaporative humidifiers, which are most frequently used at home, were selected for the experiment. These humidifiers can be remotely controlled to minimize the changes in the PM_2.5_ concentration by operating the equipment. The maximum power consumptions of the ultrasonic and natural evaporative humidifiers are 38 and 8 W, respectively.

After 10 min of starting the experiment, the humidifiers were operated at their maximum performance for 90 min via remote control, and the recording of the equipment was completed 60 min after the completion of the operation. The inside of the humidifiers was cleaned with distilled water and dried between experiments, and all the measurement intervals were set at 10 s.

In this study, 5 different water types were used to analyze the change in the indoor PM_2.5_ concentration emitted from the humidifiers: 2 types of tap water (Seoul and Gwangju, South Korea), mineral water, purified water, and distilled water. Here, the remaining types of water, except distilled water, are drinking water generally used in households in South Korea. The mineral content of each sample was measured via the ion-coupled plasma-optical emission spectroscopy (ICP-OES) analysis method, and the measurement contents were calcium (Ca), potassium (K), magnesium (Mg), and sodium (Na), as shown on the bottle of the mineral water. Moreover, regardless of the list of contents, lead (Pb) was analyzed to examine its harmfulness to the human body. Table 3 presents the measurement results of the contents for each water type, which indicated that the mineral content of tap water varies depending on the catchment area. For the purified water, most minerals contained in the tap water (Seoul, South Korea) were filtered through a nano-trap filter, which resulted in the second-lowest content following distilled water. Meanwhile, in the distilled water, no measurement contents were detected.

### 2.2. Basic Principle of the Calibration Method

As mentioned in Section 2.1, this study analyzed how the water types and humidification methods changed the PM_2.5_ concentration by continuously measuring various points simultaneously. Therefore, before starting the experiment, the precision of each equipment must be checked.

Meanwhile, TES-5322 provides calibration options for 2 points on the basis of user definition. Because AM510, which was frequently used in previous studies, is assumed to exhibit high accuracy, calibration was performed to secure the precision of TES-5322 for AM510 [29,30]. To perform the calibration, conditions to estimate the measuring range of the target PM and to maintain the concentration are required. Therefore, in this study, the analysis of the reliability of the PM_2.5_ concentration was conducted by developing and verifying a calibration system that maintains the PM_2.5_ concentration in a small chamber.

With regard to the equilibrium equations of the PM_2.5_ concentration in the steady state in a small chamber, this study used Equation (1), which was changed from the concentration equation for indoor pollutants in a steady state, and Equation (2), which is used to calculate the final state value of the mixture at by-pass by using the mixing ratio of each content [23,24,25]. In other words, the amount of the generated PM and the concentration emitted from the humidifier can be calculated using Equations (1) and (2) if the exhaust airflow rate of the chamber, PM_2.5_ concentration flowing into the chamber, and concentration of the calibration points are known. The exhaust airflow rate that is required to maintain the concentration of the calibration points can be calculated by substituting these values for the variables of each equation:

(1) $$C_{c}=C_{i}+\frac{G}{Q_{e}}$$

(2) $$C_{c}=\frac{C_{i}Q_{i}+C_{h}Q_{h}}{Q_{1}+Q_{h}},\;Q_{1}+Q_{h}=Q_{e},\;C_{h}Q_{h}=G,$$

where $C_{c}$ denotes the PM_2.5_ concentration at the calibration point (μg/m^3^); $C_{i}$, the PM_2.5_ concentration after passing through filter (μg/m^3^); G, the generation rate of the PM of the humidifier (μg/s); $Q_{e}$, the exhaust airflow rate (m^3^/h); $Q_{i}$, the supply airflow rate (m^3^/h); $C_{h}$, the PM_2.5_ concentration emitted from the humidifier (μg/m^3^); and $Q_{h}$, the humidifier airflow rate (m^3^/h).

**Figure 2:**
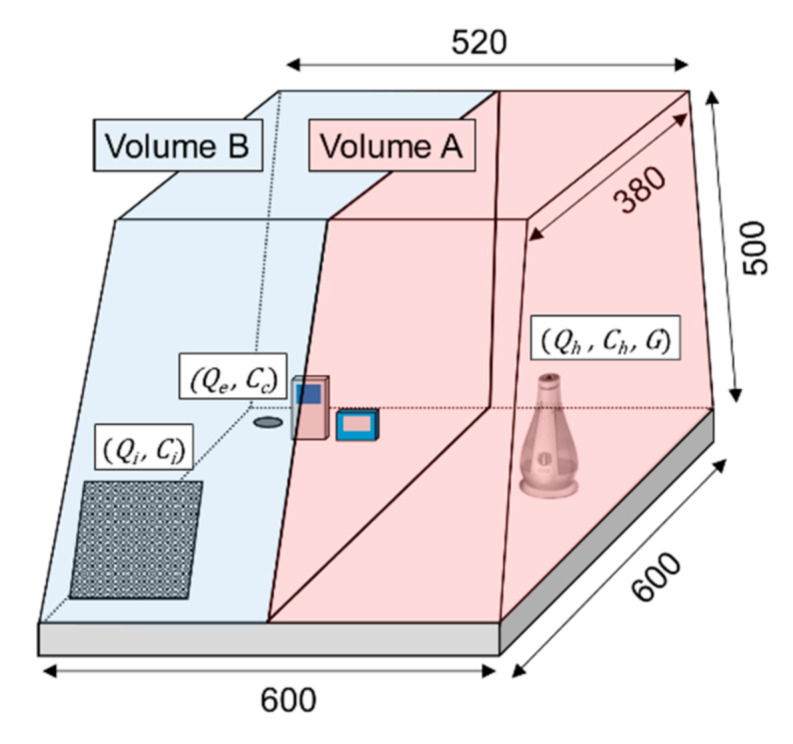
Variables of the calibration method.

Each variable of the equations above is expressed in a graphical form in Figure 2. Here, the PM_2.5_ concentration at the calibration points can be determined according to the impact made by volume B, which reduces the PM_2.5_ concentration, and volume A, which increases the PM_2.5_ concentration. Therefore, as the exhaust airflow rate increases, the external air of the chamber flowing though the filter more significantly impacts the calibration points, thus reducing the PM_2.5_ concentration of that point. In this study, calibration was conducted at 2 points below 50 μg/m^3^ and above 400 μg/m^3^. This is in accordance with the calibration replicate guidelines provided by the TESS-5322 equipment.

### 2.3. Calibration Method of the PM Measurements Using a Small Chamber

**Figure 3:**
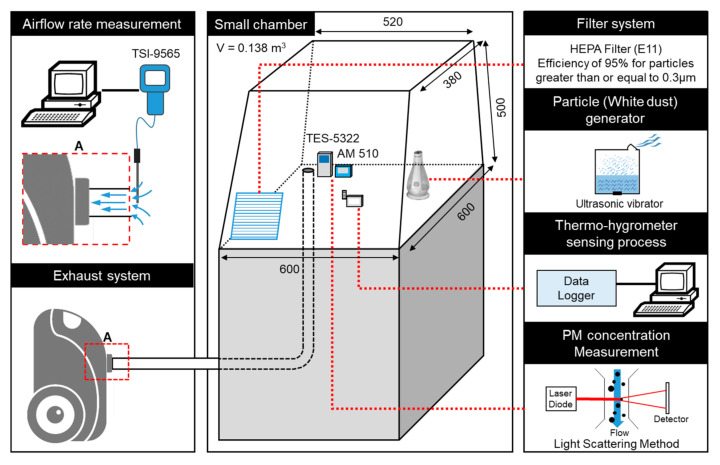
Schematic diagram of the calibration system using a small chamber.

Figure 3 presents a schematic diagram of the calibration system using a small chamber. It consisted of a small chamber, airflow rate measurement, PM_2.5_ concentration measurement, thermo-hygrometer sensing processes, exhaust system, filter, and a particle generator. In Table 4, the specifications of the experimental apparatus for equipment calibration are presented.

In this study, an experiment was conducted to measure the PM_2.5_ concentration emitted from humidifiers, and the small ultrasonic humidifier was selected as the PM generator. The advantages of an ultrasonic humidifier are its cost-effectiveness and its ability to generate a constant volume.

The space wherein calibration was performed was a small chamber made of plastic used for creating a model, with the volume of the chamber being 0.138 m^3^. TES-5322 was the equipment used to measure the PM_2.5_ concentration emitted from the humidifier, and AM510 was considered as the target for calibration of TES-5322.

**Figure 4:**
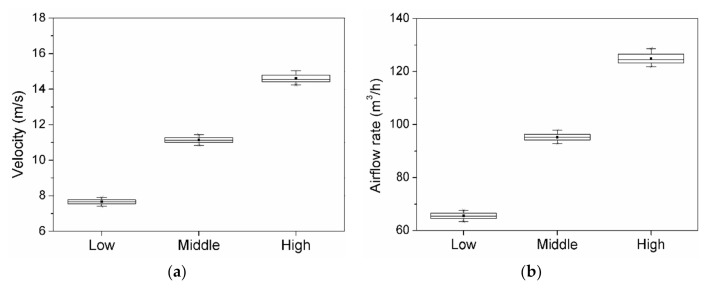
Box plot of the velocity and airflow rate: (**a**) velocity; (**b**) airflow rate.

The exhaust system was an industrial cleaner WD 5P (Karcher, Inc. Winnenden, Germany), and the exhaust velocity and volume were measured every 10 s for a total of 60 times using wind and airflow meters (TSI 9565, TSI, Inc. Shoreview, MN, USA). The measurement results are presented in Figure 4. By controlling the exhaust volume in three stages, namely, high, middle, and low, the standard deviations of the exhaust velocity and volume were 0.19 m/s and 1.63 m^3^/h, respectively, which indicates that the exhaust airflow rate was constantly maintained in high mode.

An ultrasonic humidifier, with a capacity of 180 mL, was used as the PM generator, and mineral water was selected as a sample, under the condition that a constant concentration must be maintained regardless of the period required to collect samples and the measuring range (0 to 500 μg/m^3^) of TES-5322 used in this study.

The calibration procedure was as follows. First, the calibration system of the PM measuring equipment was created. Then, the humidifier and exhaust system were turned on. When the concentration value of AM510 reached a steady state, the user option of TES-5322 was activated, and the current concentration value was input by the user.

## 3. Results and Discussion

### 3.1. Calibration Result of the PM Measuring Equipment

**Figure 5:**
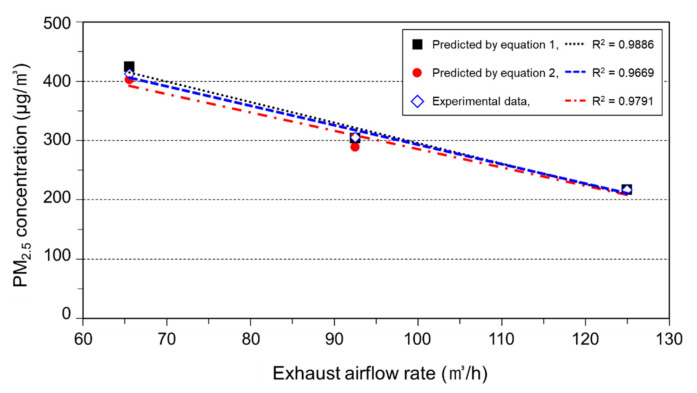
Comparison of experimental data and predicted value.

Figure 5 presents a graph of concentration $C_{c}$ at the calibration points comparing the values measured via the experiment and estimated using Equations (1) and (2) under the same conditions with changes in the exhaust airflow rate. Equation (2) has an error range of less than ±8%, and Equation (1), which estimates a homogeneous diffusion, has less than ±3%. Therefore, Equation (1) was considered to be efficient for calculating the exhaust airflow rate to create the calibration concentration by estimating the PM_2.5_ concentration $C_{i}$ flowing into the chamber through the filters, concentration of the calibration point $C_{c}$, and exhaust airflow rate $Q_{e}$. However, to create a low concentration of 50 μg/m^3^ using the calibration method employed in this study, more than 700 m^3^/h of exhaust airflow rate was required. Calibration was performed at least twice near the upper and lower limits of the concentration to be measured, and the air from the outside only was provided through filters after halting the humidifier operation to maintain the concentration at the lower limit. This was because the filter utilized in this study was of low cost, usually being installed in vehicles, and low efficiency; therefore, the PM_2.5_ concentration emitted from the filters was constantly maintained at 12–14 μg/m^3^, which was less than 50 μg/m^3^ during the experiment.

To confirm the validity of the calibration method for the PM measurement, which was developed in this study, we analyzed the concentration change during the calibration, measured the PM_2.5_ concentration for AM510 and TES-5322 under the condition of varying PM_2.5_ concentrations in the same space, and analyzed the measured values statistically and visually.

**Figure 6:**
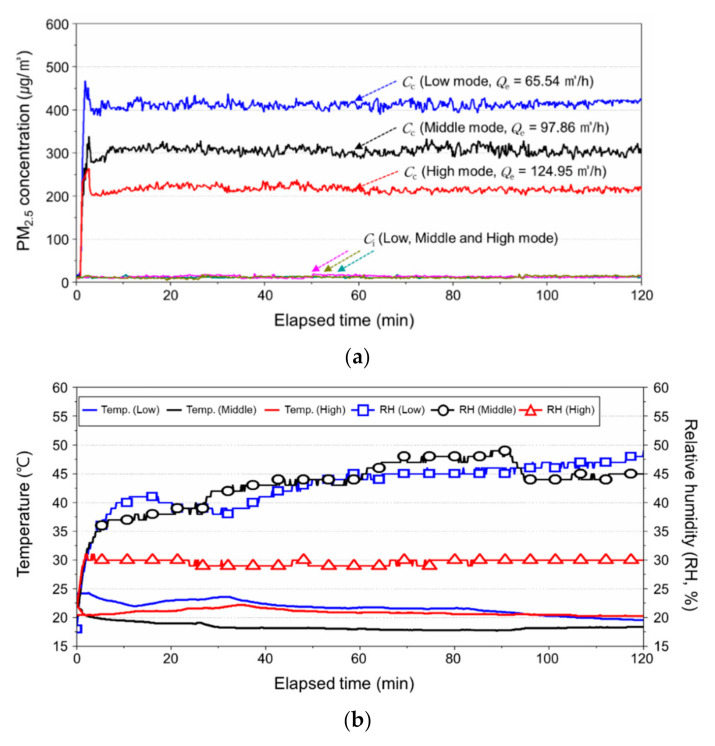
PM_2.5_ concentration, temperature, and relative humidity at the measurement point during calibration: (**a**) PM_2.5_ concentration; (**b**) temperature and relative humidity.

Figure 6 presents the change in temperature, relative humidity, and PM_2.5_ concentration at the calibration points. As can be seen from the figure, the PM_2.5_ concentration at the calibration points decreased as the exhaust airflow rate increased, and the PM_2.5_ concentration reached a steady state 20 min after operating the exhaust system and humidifier and remained steady afterwards. Accordingly, the average of PM_2.5_ concentration per three control mode via 541 points of data measured every 10 s from 30 to 60 min after operating the system were 413, 305, and 216 μg/m^3^, respectively, and the standard deviations were 8, 8, and 6 μg/m^3^, respectively. Considering that the accuracy of AM501 in the PM measurement was ±10% at the stages higher than 50 μg/m^3^, the accuracy was found to be very high.

Meanwhile, the temperature and humidity were analyzed to check whether the relative humidity exceeded 95% of the maximum operation value of the equipment when using humidifiers in a small chamber. On the basis of the results, the maximum value of the relative humidity was less than 50%. This indicates that calibration using a small chamber and a humidifier can maintain the relative humidity within the operation range if the exhaust airflow rate was sufficiently high.

**Figure 7:**
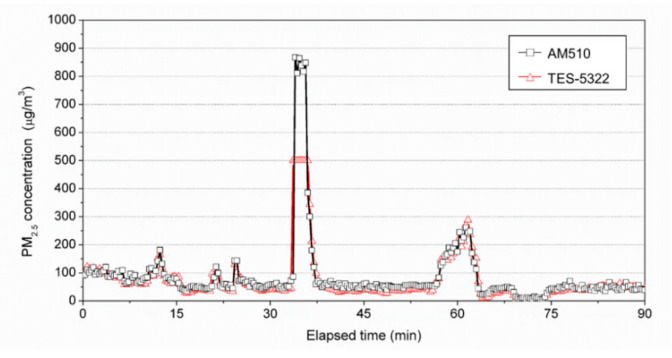
Comparison of the PM_2.5_ concentration measurement results of AM510 and TES-5322 after calibration.

AM510 and the calibrated TES-5322 were installed under the condition of varying PM_2.5_ concentrations to measure the PM_2.5_ concentration every 10 s. The measurement results are presented in Figure 7. The values of the two equipment were similar, even in the range of increase or decrease in the PM_2.5_ concentration.

Moreover, the correlation coefficient (*ρ*) was found to be 0.964, indicating that the concentration values of the two equipment became similar via calibration, excluding the upper measurement range limit (above 500 μg/m^3^) of the TES-5322.

### 3.2. Change in the Indoor Environment by Using Ultrasonic Humidifiers

#### 3.2.1. Change in the Indoor PM_2.5_ Concentration

After checking the amount of water used for humidifiers before and after the experiment, we found the amount of humidification to be about 300 ± 10 mL/h, regardless of types of water.

**Figure 8:**
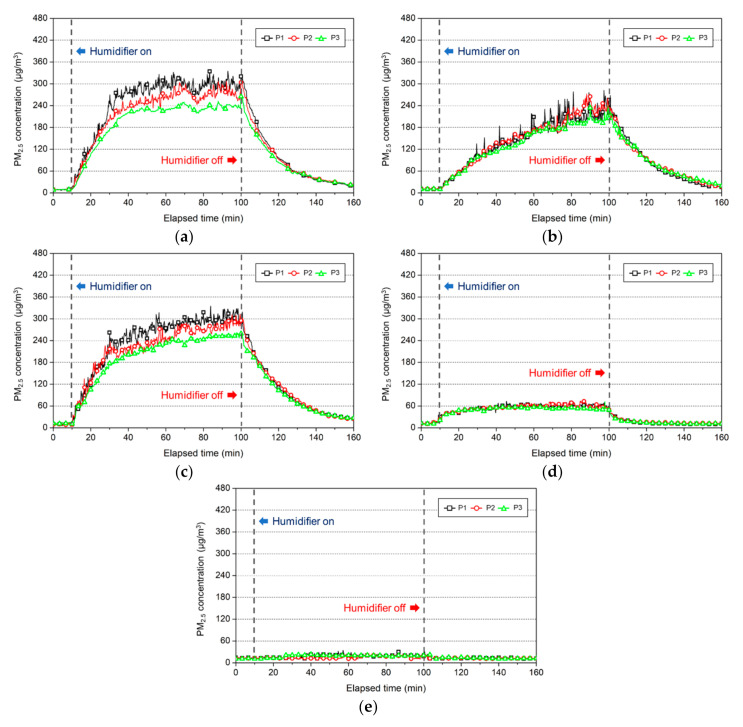
PM_2.5_ concentration according to the use of an ultrasonic humidifier for each water type: (**a**) tap water (Seoul); (**b**) tap water (Gwangju); (**c**) mineral water; (**d**) purified water; (**e**) distilled water.

Ultrasonic humidifiers were operated using five different types of water, and the results as shown in Figure 8, revealed that the indoor PM_2.5_ concentration after 90 min was high in the order of mineral water, tap water (Seoul), tap water (Gwangju), purified water, and distilled water. The PM_2.5_ concentration rapidly increased for the three water types, excluding purified and distilled water, immediately after the ultrasonic humidifier was operated, and measuring point 3, which was 2.55 m away from the humidifier, exhibited a similar increase. Meanwhile, Yao et al. (2020) analyzed the size-resolved particle number concentration of PM emitted from an ultrasonic humidifier and confirmed that the lowest concentration was observed at the farthest measuring point. This is similar to the result of the present study, which demonstrated the lowest PM_2.5_ concentration at the farthest measuring point [13].

The PM_2.5_ concentration was found to be a maximum of 350 μg/m^3^ (tap water, Seoul). This value was very high, considering that the appropriate indoor PM_2.5_ concentration for 24 h proposed by the U.S. National Ambient Air Quality Standards (NAAQS) is 35 μg/m^3^ or less [31].

The size of the experimental space was 31.5 m^3^, indicating that a high PM_2.5_ concentration can be generated according to the water types used for an ultrasonic humidifier in a small space.

Moreover, even if tap water was used, the indoor PM_2.5_ concentration would vary depending on the place where the water was obtained.

With regard to distilled water, it had little or no impact on the increase in the PM_2.5_ concentration, despite the use of an ultrasonic humidifier. This was because the water particles decomposed by the ultrasonic oscillator were smaller than the lower detection limit of the measurement equipment (0.1 μm) or evaporated before reaching the measuring point, thus preventing the detection of the PM_2.5_ concentration.

Meanwhile, the concentration rapidly decreased, regardless of the PM_2.5_ concentration range, immediately after halting the humidifier operation, and 1 h later, the concentration level before the experiment was reached. This can seen to be the differentiating characteristic between the white dust and other particulate pollutants.

**Figure 9:**
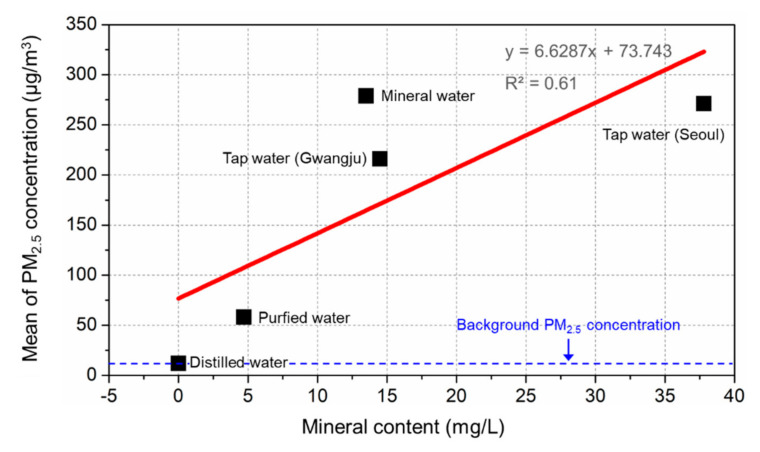
Comparison of the mean of the PM_2.5_ concentration and mineral content.

Therefore, in this study, the correlation between the mean of the PM_2.5_ concentration over 80–100 min at three measuring points and four water types of content was determined to identify what caused the generation of concentration for each sample used for the humidifier, and the results are presented in Figure 9.

As can be seen from the figure, as the mineral content of a sample increased, the mean of the PM_2.5_ concentration over 80–100 min at three measuring points also increased. However, the correlation coefficient was analyzed to be 0.61. Moreover, even though the mineral content was almost identical between tap water (Gwangju) and mineral water, the variation of the mean of the PM_2.5_ concentrations was more than 20%. Therefore, to quantitatively evaluate the impact of mineral on the generation of the PM_2.5_ concentration by white dust, the contents in addition to the four types of minerals, including TDS, and analysis of the impact made by each mineral were required [15].

#### 3.2.2. Change in the Temperature and Relative Humidity

**Figure 10:**
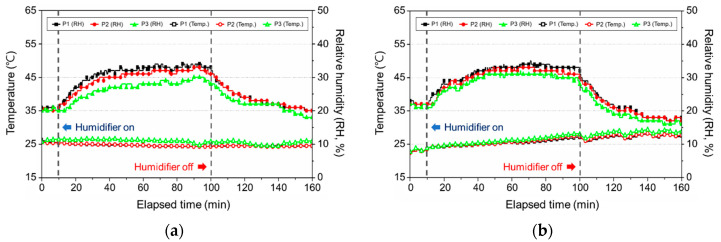

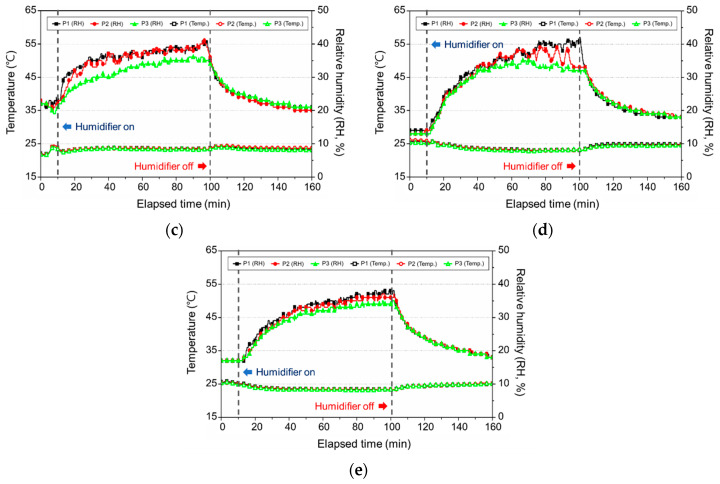
Temperature and relative humidity based on the use of an ultrasonic humidifier for each water type: (**a**) tap water (Seoul); (**b**) tap water (Gwangju); (**c**) mineral water; (**d**) purified water; (**e**) distilled water.

To analyze the maintenance of indoor humidity, we compared the temperature and relative humidity, with the results being presented in Figure 10. As can be seen from the figure, even though the total amount of used water, while operating the ultrasonic humidifier for 90 min at its maximum performance, was 450 mL, the indoor relative humidity was mostly 40%. This was because the experimental space had a gypsum tex, which caused extensive water absorption into the ceiling. Saint et al. (2017) conducted a study on relative humidity according to the operation of the ultrasonic humidifier for 8 h in an experimental space measuring 33.4 m^3^, which was similar to the space used in the present study. In the experiment, a relative humidity of 29% was measured, which was much lower than the predicted value of 77.6% of the relative humidity. It can be inferred that the carpet spread around the humidifier absorbed the humidity. This was similar to the result of the present study, which demonstrated that the relative humidity was 40% instead of the predicted value of 80% [15]. This showed that if the humidifier is operated in an auto mode when the finishing materials are dry or infiltration is high, then an appropriate humidity cannot be maintained, and even the PM_2.5_ concentration, including white dust, could increase. Therefore, when the ultrasonic humidifier is to be operated in such an environment, it is determined that an increase in the PM_2.5_ concentration can be minimized by using purified water or distilled water.

### 3.3. Change in the Indoor Environment by Using a Natural Evaporative Humidifier

#### 3.3.1. Change in the Indoor PM_2.5_ Concentration

**Figure 11:**
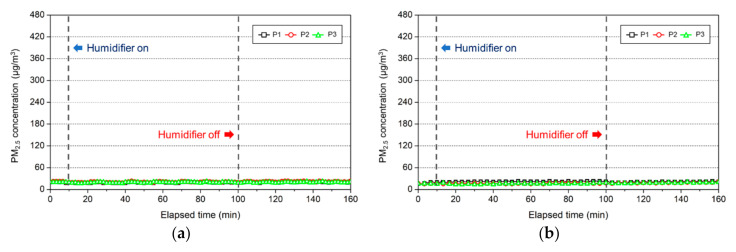
PM_2.5_ concentration according to the use of a natural evaporative humidifier for each water type: (**a**) tap water (Seoul); (**b**) distilled water.

The change in the PM_2.5_ concentration resulting from the use of a natural evaporative humidifier was analyzed for each sample, and the results are presented in Figure 11. Distilled water was selected as the comparison group, whereas tap water from Seoul was selected as the sample as it is the most widely used and exhibited high concentrations in the ultrasonic humidifier experiment. The use of tap water (Seoul) is very reasonable and economical, as 1000 L of water only costs USD 0.31 (October 2020, Seoul, South Korea).

According to the PM measurements, tap water (Seoul), which exhibited a PM_2.5_ concentration of 300 μg/m^3^ in the ultrasonic humidifier experiment, generated PM_2.5_ concentration at the background concentration level or exhibited almost no increase in the concentration level when a natural evaporative humidifier was used, as was the case for distilled water. This was because the PMs emitted from a natural evaporative humidifier in the form of aerosol are smaller than the detection limit of the equipment or completely evaporate, as analyzed in the other study [15].

#### 3.3.2. Change in the Temperature and Relative Humidity

**Figure 12:**
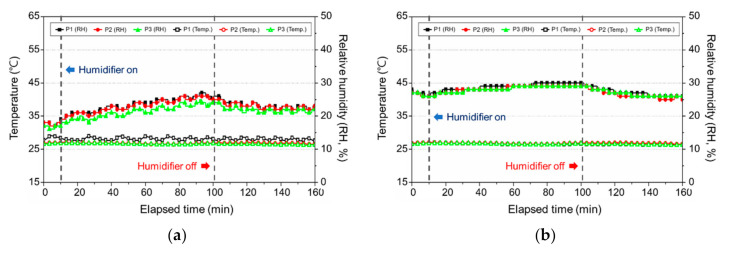
Temperature and relative humidity according to the use of a natural evaporative humidifier for each water type: (**a**) tap water (Seoul); (**b**) distilled water.

Figure 12 presents the change in the indoor temperature and humidity by using a natural evaporative humidifier. The △RH of the ultrasonic humidifier, which is the difference in the relative humidity before and after operation, was 17% on average, but the ∆RH of the natural evaporative humidifier was 8%, indicating a difference in the performance on the basis of the humidification method. Thsu, a 2015 consumer report in the USA, revealed that ultrasonic humidifiers were mostly favored because their humidification ability is good [31].

## 4. Conclusions

This study analyzed the change in the indoor PM_2.5_ concentration on the basis of the water types and humidification methods used at home, and the results are summarized as follows.

Aerosol-type particulate pollutants were generated using humidifiers; a calibration system was installed using a small chamber by controlling the exhaust airflow rate, and the measurement intensity of the target and subject equipment were unified. After the calibration was completed, the error range of the concentration was less than 3% between the forms of equipment, thus suggesting its potential for high levels of utilization in relevant studies in the future, taking into consideration the cost of system installation.

In the case of using a natural evaporative humidifier, no increase in the indoor PM_2.5_ concentration was observed, regardless of the water types. Meanwhile, the concentrations for the same tap water significantly varied on the basis of the water supply system. With regard to the tap water used in this study, more than 200 contents were thoroughly checked on the basis of the Korean water supply laws, and no problems were observed with the drinking of such water.

Meanwhile, the indoor PM_2.5_ concentration was reduced to the background level 30 min after halting the ultrasonic humidifier operation, regardless of the sample; thus, it is recommended that one first turn on the humidifier in advance, let the room reach an appropriate humidity range, and then turn off the humidifier and enter the room after at least 20–30 min. As an alternative, water purified with nano-trap filters must be utilized for ultrasonic humidifiers, and the use of natural evaporative humidifiers is recommended to reduce the generation of the indoor PM concentration, regardless of the sample.

While four mineral contents contained in the water were investigated in this study, contents other than pure minerals may impact the generation of PM_2.5_ concentration due to the use of an ultrasonic humidifier. Therefore, to identify their relevant contents and examine their harmfulness to the human body, further research is required.

## Figures and Tables

**Table 1:** Specifications of the particulate matter (PM)_2.5_ measuring instrument used in this study.

Parameter		AM510	TES-5322
Measurement		90° light scattering	Light scattering
Sensor type		670 nm laser diode	-
Particle size range (μm)		0.1 to 10	0.1 to 2.5
Concentration range (μg/m^3^)		0.1 to 20,000	0.1 to 500
Accuracy	≤50 μg/m^3^	-	±5 μg/m^3^
	>50 μg/m^3^	-	10%
Operational condition	Temperature (°C)	0 to 50	0 to 60
	RH (%)	0 to 95	0 to 95
	Capacity	Approximately *N* = 31,000	MicroSD card 4 GB
Price (USD, $)		Approximately 3800	Approximately 400

**Table 2:** Specifications of two types of humidifiers.

Parameter	Value	
	Smartmi Air Humidifier	Smartmi Air Humidifier 2
Type	Ultrasonic	Natural evaporative
Capacity (L/h)	0.355	0.24
Tank capacity (L)	3.5	4
Noise level (dB)	Approximately 40.4	Approximately 34.3
Energy use (W)	38	8
Wireless technology	802.11 b/g/n, 2.4 GHz	

**Table 3:** Mass concentration of mineral in each water type (unit: mg/L).

Water Type	Calcium (Ca)	Sodium (Na)	Potassium (K)	Magnesium (Mg)	Lead (Pb)
Tap water (Seoul)	20.1	2.8	3.8	11.1	None
Tap water (Gwangju)	7.3	1.6	1.9	3.7	None
Mineral water	3.2	2.5	2.8	5.0	None
Purified water	1.8	0.5	0.4	2.0	None
Distilled water	None	None	None	None	None

**Table 4:** Specifications of the experimental apparatus for equipment calibration.

Component	Model	Parameter	Value
Airflow Measurement	TSI 9565	Range (m/s)	0 to 50
		Accuracy (m/s)	±0.015
PM measurement	AM510/TES-5322	Table 1	
Temperature and RH measurement	TR-72WF	Range (°C and %)	0 to 55 and 10 to 95
		Accuracy (°C and %)	±0.5 and ±5
		Resolution (°C and %)	0.1 and 1
Exhaust system	WD 5 P	Energy use (W)	1100
		Container capacity (L)	25
		Dimensions (mm)	418 × 382 × 652
Filter	GFAH-1005	Efficiency (%)	98 (0.5 to 1.0 μm)
		Size (mm)	175 × 170
Particle generator	DP-2030UH	Humidification type	Ultrasonic
		Capacity (L/h)	0.18
		Tank size (L)	1
